# Benefits of simultaneous laparoscopic colorectal surgery and liver resection for colorectal cancer with synchronous liver metastases: Retrospective case-matched study

**DOI:** 10.1016/j.amsu.2020.09.009

**Published:** 2020-09-06

**Authors:** Wipusit Taesombat, Prapon Kanjanasilp, Bunthoon Nonthasoot, Methee Sutherasan, Athaya Vorasittha, Boonchoo Sirichindakul

**Affiliations:** Department of Surgery, Faculty of Medicine, King Chulalongkorn Memorial Hospital, Chulalongkorn University, Bangkok, Thailand

**Keywords:** Colorectal cancer, Liver metastases, Laparoscopic surgery, Simultaneous surgery

## Abstract

**Introduction:**

Laparoscopic surgery for colorectal cancer and liver tumors are accepted as alternative procedure to open surgery. However, few studies reported outcomes of simultaneous laparoscopic surgery of these two procedures. The aim of this study was to compare short-term outcomes between laparoscopic and open approach.

**Materials and methods:**

Between June 2010 to December 2019, simultaneous laparoscopic cases were retrospectively matched (1:2) to open cases. Peri-operative and short-term outcomes were compared between both groups.

**Results:**

Twelve patients in laparoscopic group were matched to 24 patients in open group according to age, gender, body mass index, american society of anesthesiologists physical status, preoperative laboratory data, number and size of liver metastases and extent of colorectal and liver resection, Most patients in each group had left-sided colon or rectal cancer and underwent wedge liver resection. The mean number of liver metastases was 1.3 vs 1.5 and size of liver metastases was 2.2 ± 1.4 vs 2.7 ± 1.1 cm in laparoscopic compared to open group. Estimated blood loss and length of hospital stay were significantly lower in laparoscopic group. However, operative time was significantly longer in laparoscopic group. Peri-operative complication was not significant difference between both groups and there was no mortality.

**Conclusion:**

Simultaneous laparoscopic colorectal surgery and minor liver resection is feasible and safe. Laparoscopic approach has better peri-operative outcome in term of shorter length of hospital stay compared to open approach.

## Highlights

• Outcome of simultaneous laparoscopic surgery for colorectal cancer with synchronous liver metastases remains controversy.
• This study showed comparable peri-operative outcomes of simultaneous laparoscopic compared to open surgery.
• Simultaneous laparoscopic surgery had shorter length of hospital stay and less operative blood loss.

## 1 Introduction

Colorectal cancer (CRC) is the second most common cause of cancer related death worldwide [1], 15–20% of patients have liver metastases at the time of diagnosis [2]. The curative surgery is the only chance of long-term survival [3]. There are various treatment strategies for colorectal cancer with synchronous liver metastases. First, classical two-stage procedure with resection of primary colorectal cancer and follow by subsequent liver resection or less commonly performed liver resection first.

Another treatment strategy is simultaneous approach with potential benefits of shorter hospital stay and shorter time to receive systemic treatment after surgery [4]. However, there are concerns of higher morbidities of performing two major surgery simultaneously [5]. There are studies from highly experience center that showed acceptable morbidity and mortality rate for particularly open surgery of simultaneous approach.

For patients that presented with severe symptoms of primary tumor such as complete obstruction, massive bleeding or perforation that necessitated emergency surgical treatment of colorectal cancer first. Then, subsequent liver resection was planned upon patient completed recovery. For patient with multiple bilobar liver metastases which prognosis of patient depended on liver metastases, liver first approach may be planned. In general, liver first approach is actually systemic chemotherapy first then was evaluated for liver resection. However, for patient with asymptomatic or minimal symptomatic primary tumor with limited liver metastases, each treatment strategies may be used.

Recently, minimally invasive surgery of colorectal resection and liver resection particularly minor liver resection have been accepted as standard treatment. However, few studies reported feasibility and surgical outcomes of simultaneous laparoscopic surgery of these two procedures [[6], [7], [8]].

The aim of this study was to compare peri-operative outcomes of simultaneous laparoscopic to open approach using case-matched study.

## 2 Materials and methods

### 2.1 Study design

We retrospectively reviewed medical records of patients with colorectal cancer with synchronous liver metastases who underwent simultaneous colorectal and liver resection at our university hospital between June 2010 and December 2019. Patients with colorectal cancer with synchronous liver metastases who underwent simultaneous laparoscopic resection were matched 1:2 to patients who underwent simultaneous open resection. Preoperative patient characteristic including age,gender, body mass index, American Society of Anesthesiologists (ASA) physical status, preoperative laboratory data, primary colorectal cancer location, liver tumor diameter and number of liver tumor were used to select patients to match between both groups. There were 155 patients who underwent simultaneous surgery during this study period. Twelve patients in laparoscopic group were matched to 24 patients in open group.

The treatment plan was discussed in multidisciplinary conference including hepatobiliary surgeons, interventional radiologists, diagnostic radiologists and medical oncologists. In general, patient with ability to achieve curative resection, good performance status and well preserved liver function can be candidate for simultaneous surgery. The choices between laparoscopic and open surgery depended on surgeons preference. Extent of planned liver resection was not modified by using the laparoscopic approach. The peri-operative data including preoperative data (demographic data, laboratory and radiologic evaluation), operative data (procedure, blood loss, blood transfusion requirement) and postoperative data (laboratory change, hospital stay, complications) were collected.

Postoperative morbidity was defined as events that occurred during the first 60 days after surgery. Complications were graded by using Clavien-Dindo classification [9]. Postoperative mortality was defined as death within 90 days after surgery.

### 2.2 Preoperative evaluation

All patients underwent laboratory evaluation including complete blood count, creatinine, prothrombin time, activated partial thromboplastin time, international normalized ratio, liver function test, carcinoembryonic antigen (CEA) and chest radiograph, and preoperative consultation as needed. The diagnosis and staging of colorectal cancer were obtained by colonoscopy and triple-phase CT scan or contrast-enhanced MRI of abdomen.

### 2.3 Surgical procedures

Type of liver resection was planned according to tumor location for achieving negative resection margins. For colorectal resection was performed according to standard procedures. Most patients underwent liver resection first and then colorectal resection, however the sequence of procedure may altered according to availability of surgeons.

For open surgery, patient was placed in supine or lithotomy position based colorectal procedures. Long midline incision was used. General intraabdominal exploration for extrahepatic metastatic disease. For liver resection, intraoperative ultrasonography was performed routinely for tumor identification and marking resected line. Pringle's maneuver was prepared for all patients. Parenchymal transection was performed by using electrocauterization in combined with Cavitron Ultrasonic Surgical Aspirator (CUSA, Valley Lab Inc., CO, USA). For colorectal resection, standard procedure was performed. Bowel anastomosis was created using stapler technique. For low rectal anastomosis, protective colostomy/ileostomy was performed.

For Laparoscopic surgery, patient was placed in lithotomy position. Umbilical incision was performed by open technique for creating pneumoperitoneum with pressure 8–12 mmHg and camera insertion. Then additional 3–4 ports were placed for liver resection. Pringle's maneuver was prepared in all patients. Parenchymal transection was performed by using CUSA, Harmonic scalpel (Ethicon Endo-Surgery Inc.), surgical clips and vascular stapler. For colorectal resection, additional 2–3 ports were placed according to planned procedure. Technique of bowel anastomosis and use of protective ostomy were similar to open surgery. The resected specimen was placed in plastic bag and removed through extended umbilical incision or Pfannelstiel incision. Postoperative tube drainage for both open and laparoscopic resection was routinely placed.

### 2.4 Postoperative care and follow up

Postoperatively, all patients underwent daily examination by surgeons and laboratory evaluation including complete blood count, serum creatinine and liver function test. Drain was removed when drainage fluid was serous, no evidence of bile leakage and less than 100 ml/day in volume. After discharge, all patients were followed up every 3 months for the first 2 years and then every 4–6 months. For each follow up visits, complete blood count, liver function test, serum CEA and triple phase CT scan or contrast-enhanced MRI of abdomen were obtained.

### 2.5 Statistical analysis

Patient characteristics were described using mean ± standard deviation. We compared patient characteristics using unpaired t-tests and nonparametric analysis as appropriate. All analyses were performed in Stata 13 (StataCorp LP, College Station, TX), and *p*-values less than 0.05 were considered statistically significant. The work has been reported in line with the STROCSS criteria [10]. The research registration unique identifying number is researchregistry5947 (hyperlink: https://www.researchregistry.com/register-now#user-researchregistry/registerresearchdetails/5f4719f0758c810015b6e320/).

## 3 Results

### 3.1 Baseline characteristics

Between June 2010 and December 2019, there were 140 patients with colorectal cancer with synchronous liver metastases who underwent simultaneous surgery at our institution. Twelve patients who underwent laparoscopic surgery were matched (1:2) to 24 patients who underwent open surgery. Baseline patient characteristics were not significant difference between both groups (Table 1). More than two-third of patients had primary left sided colon or rectal cancer. Most patients had number of liver metastases less than 3 and maximal tumor diameter less than 3 cm. Location of liver metastases were mostly in peripheral segment (segment II, III, IV lower and VI) in both group. Only one tumor in laparoscopic group and 3 tumors in open group located in segment VII or VIII. Few patients with rectal cancer in each group underwent preoperative radiation.

**Table 1:** Baseline characteristics of patients who had laparoscopic or open surgery.

Characteristics	Laparoscopic group (n = 12)	Open group (n = 24)	*p* value
Age(years)	69.4 ± 9.1	63.3 ± 12.3	0.10
Gender (M/F)	6/6	13/11	0.82
BMI (kg/m^2^)	22.9 ± 4	23.8 ± 3.7	0.56
ASA classification			0.65
class I	6 (50.0)	14 (58.3)	
class II	6 (50.0)	10 (42.7)	
Tumor diameter (cm)	2.2 ± 1.4	2.7 ± 1.1	0.31
Number of liver metastases	1.3 ± 0.5	1.5 ± 0.7	0.13
Primary tumor site			0.06
Right colon	3 (25.0)	3 (12.5)	
Left colon	5 (41.7)	8 (33.3)	
Rectum	4 (33.3)	13 (54.2)	
Preoperative chemotherapy	2 (16.7)	4 (16.7)	
Preoperative laboratory			
Hematocrit (%)	33.5 ± 3.7	37.5 ± 5.1	0.34
White blood cell (*1000/mm^3^)	6.5 ± 2.2	7.4 ± 2.9	0.33
Platelets (*1000/mm^3^)	261 ± 148	295 ± 108	0.49
Total bilirubin (mg/dl)	0.4 ± 0.2	0.6 ± 0.3	0.17
AST (IU/L)	21.3 ± 8.4	20.9 ± 7.5	0.90
ALT (IU/L)	16.3 ± 8.6	17.3 ± 9.3	0.76
Albumin (mg/dl)	3.5 ± 0.9	3.8 ± 0.5	0.28
Creatinine (mg/dl)	0.9 ± 0.2	0.9 ± 0.2	0.91
INR	1.0 ± 0.1	1.1 ± 0.1	0.39
CEA at diagnosis (ng/dl)	21.2 ± 39.3	137.9 ± 269.5	0.05

Data are shown as number (%) or mean ± standard deviations BMI, Body mass index.

ASA, American society of Anesthesiologists physical status; AST, aspartate aminotransferase; ALT, alanine aminotransferase; INR, international normalized ratio; CEA,carcinoembryonic antigen.

### 3.2 Peri-operative outcomes

More than one-third of patients had anterior or low anterior resection and most of patients had wedge liver resection as shown in Table 2. There was no conversion in laparoscopic group. The operative time was longer in laparoscopic group. Blood loss was lower in laparoscopic group, however number of blood transfusion was not difference between both groups. Postoperative complication was not difference between both groups. There was no postoperative mortality in both groups. Laparoscopic group had significant shorter length of hospital stay compared to open group.

**Table 2:** Peri-operative outcomes of patients who had laparoscopic or open surgery.

Characteristics	Laparoscopic group (n = 12)	Open group (n = 24)	*p* value
Type of colorectal resection			0.06
Right hemicolectomy	3 (25.0)	3 (12.5)	
Left hemicolectomy	4 (33.3)	5 (20.9)	
Anterior/low anterior resection	4 (33.3)	12 (50.0)	
Total abdominal colectomy	1 (8.4)	2 (8.3)	
Posterior exenteration	0	2 (8.3)	
Type of liver resection			0.52
Wedge resection	10 (83.3)	22 (91.7)	
Lateral sectionectomy	2 (16.7)	2 (8.3)	
Operative time (minutes)	494.6 ± 129.4	313.8 ± 80.9	<0.001
Blood loss (ml)	291.7 ± 181.9	497.9 ± 329.2	0.04
Blood transfusion	2 (16.7)	5 (20.8)	0.14
Postoperative complication	4 (33.3)	10 (41.7)	0.64
Grading of complication			
Grade I	1	1	
Grade II	2	1	
Grade IIIa	1	7	
Grade IIIb	0	1	
Hospital stay (days)	8.2 ± 4.6	16.8 ± 13.0	0.007
Time to first postoperative chemotherapy (days)	49.8 ± 17.1	53.4 ± 23.3	0.67

Data are shown as number (%) or mean ± standard deviations.

Number of patient who had negative resection margins was not difference between both groups as shown in Table 3.

**Table 3:** Pathological results of patients who had laparoscopic or open surgery.

Characteristics	Laparoscopic group (n = 12)	Open group (n = 24)	*p* value
T stage of primary tutor			
T3/T4	8/4	20/4	0.32
Node positive	10 (83.3)	18 (75.0)	0.57
LVI positive	10 (83.3)	13 (54.2)	0.06
Degree of differentiation			
well/moderate/poor	5/6/1	13/11/0	0.35
Negative resection margin	11 (91.7)	20 (83.3)	0.66

Data are shown as number (%); LVI, Lymphovascular invasion.

## 4 Discussion

There were three options regarding surgical treatment of colorectal cancer with synchronous liver metastases including classical two-stage procedure, liver first approach and simultaneous approach. In general, each treatment options showed no different in long-term overall survival and peri-operative complications [11, 12]. However, proper selection of treatment depended on several factors such as symptom of primary tumor, extent of liver metastases, performance status of patient and institutional policy. Simultaneous approach may offer benefits of shorter hospital stay and shorter overall time to receive systemic treatment with acceptable postoperative complication rate [4,5,12].

Laparoscopic liver resection had steep and long learning curve [[13], [14], [15]] resulted in slower development when compared to other laparoscopic procedures, particularly colorectal surgery. Recent international consensus guideline recommended that laparoscopic minor liver resection and lateral sectionectomy were standard treatment [16]. However, peri-operative morbidities of this combined laparoscopic colorectal and liver resection remained controversial. Tranchart et al. [7] and Wei et al. [17] showed comparable postoperative complication rate when compared to open surgery. This present study also showed no different in postoperative morbidity and mortality between simultaneous open and laparoscopic surgery for colorectal cancer with synchronous liver metastases. Laparoscopic surgery provided better visualization with magnification view that allowed surgeon to perform dissection precisely and meticulously. Besides, the effect of pneumoperitoneum can decrease bleeding from hepatic vein during laparoscopic liver resection was performed. Most studies showed less operative blood loss for laparoscopic compared to open liver resection [[18], [19], [20]]. This present study also showed less operative blood loss in laparoscopic group, although blood transfusion rate was not significantly different. After laparoscopic surgery, early returned of bowel function and less postoperative pain were expected resulting in shorter length of hospital stay. This study also showed benefits of simultaneous laparoscopic surgery in term of shorter hospital stay. However, this study showed longer operative time in laparoscopic group, several study showed equal or even shorter operative time, but mostly were in highly experience center [7, 21, 22]. This was the effect of learning curve in the early period, particularly for laparoscopic liver resection. In the later cases, operative time was decreasing. Most liver tumors in this study were located in peripheral segment and less than 3 cm in maximal diameter which were favorable for laparoscopic liver resection. Besides, most patients with rectal cancer did not received preoperative radiation that may indicated less complicated rectal surgery. This favorable characteristics of colorectal cancer with liver metastases may be suitable criterias for simultaneous laparoscopic surgery.

Although, this is single center study, there were some limitations of the study. First, the number of patient was small, however it represented single center experience. Most studies that reported large number of patients were multicenter study. Second, this study showed only short-term outcomes, however authors have continued to collect long-term outcomes. Third, all laparoscopic liver resections in this study were minor resection. It may be difficult to generalize outcomes for general populations. There were very few studies reported simultaneous laparoscopic major hepatectomy [23, 24] and the outcome was still debated. Lastly, this study was non-randomized controlled. Although it was matched study, there were some possibilities of selection bias.

In conclusions, this study showed that simultaneous laparoscopic colorectal surgery and minor liver resection was feasible and safe. Besides, laparoscopic approach has better short-term outcomes regarding shorter hospital stay and less blood loss compared to open approach.

## Ethical approval

The study protocol was approved by the institutional review board, faculty of medicine, Chulalongkorn university. (IRB number 336/58).

## Funding sources

No financial support was received for this study.

## Registration of research studies

Name of the registry: Benefits of simultaneous laparoscopic colorectal surgery and liver resection for colorectal cancer with synchronous liver metastases: retrospective case-matched study.

Research Registry Unique Identifying Number: researchregistry5947.

Hyperlink to the registration: https://www.researchregistry.com/register-now#user-researchregistry/registerresearchdetails/5f4719f0758c810015b6e320/

## Author contribution

Wipusit Taesombat; concept of study, data collection, data analysis, writing paper, Prapon Kanjanasilp; concept of study, data collection, Methee Suthrasan; data collection, Athaya Vorasittha; data collection, Bunthoon Nonthasoot; concept of study, data collection, Boonchoo Sirichindakul; concept of study, critical manuscript revision.

## Guarantor

Wipusit Taesombat.

## Provenance and peer review

Not commissioned, externally peer reviewed.

## Declaration of competing interest

All of authors in this study declared that there were no conflicts of interest.

## References

[1] (2019). The Global Cancer Observatory.

[2] Adam R., de Gramont A., Figueras J., Kokudo N., Kunstlinger F., Loyer E. (2015). Managing synchronous liver metastases from colorectal cancer: a multidisciplinary international consensus. Canc. Treat Rev..

[3] Chow F.C., Chok K.S. (2019). Colorectal liver metastases: an update on multidisciplinary approach. World J. Hepatol..

[4] Abelson J.S., Michelassi F., Sun T., Mao J., Milsom J., Samstein B. (2017). Simultaneous resection for synchronous colorectal liver metastasis: the new standard of care?. J. Gastrointest. Surg..

[5] Watanabe T., Itabashi M., Shimada Y., Tanaka S., Ito Y., Ajioka Y. (2015). Japanese society for cancer of the colon and rectum (JSCCR) guidelines 2014 for treatment of colorectal cancer. Int. J. Clin. Oncol..

[6] Poel M.J., Tanis J., Marsman H.A., Rijken A.M., Gertsen E.C., Ovaere S. (2019). Laparoscopic combined resection of liver metastases and colorectal cancer: a multicenter, case-matched study using propensity scores. Surg. Endosc..

[7] Tranchart H., Fuks D., Vigano L., Ferretti S., Paye F., Wakabayashi G. (2016). Laparoscopic simultaneous resection of colorectal primary tumor and liver metastases: a propensity score matching analysis. Surg. Endosc..

[8] Bizzoc C., Delvecchio A., Fedele S., Vincenti Leonardo (2019). Simultaneous colon and liver laparoscopic resection for colorectal cancer with synchronous liver metastases:A single center experience. J. Laparoendosc. Adv. Surg. Tech..

[9] Dindo D., Demartines N., Clavien P.A. (2004). Classification of surgical complications a new proposal with evaluation in a cohort of 6336 patients and results of a survey. Ann . Surg..

[10] Agha R., Abdall-Razak A., Crossley E., Dowlut N., Iosifidis C., Mathew G. (2019). The STROCSS 2019 guideline: strengthening the reporting of cohort studies in surgery. Int. J. Surg..

[11] Silberhumer G.R., Paty P.B., Denton B., Guillem J., Gonen M., Araujo R.L.C. (2016). Long- term oncologic outcomes for simultaneous resection of synchronous metastatic liver and primary colorectal cancer. Surgery.

[12] Kelly M.E., Spolverato G., Le G.N., Mavros M.N., Doyle F., Pawlik T.M. (2015). Synchronous colorectal liver metastasis: a network meta-analysis review comparing classical, combined, and liver-first surgical strategies. J. Surg. Oncol..

[13] Taesombat W., Nonthasoot B., Sutherasan M., Vorasittha A., Sirichindakul B. (2020). Assessment of learning curve for laparoscopic liver resection in low-volume center. J. Med. Assoc. Thai..

[14] Vigano L., Laurent A., Tayar C., Tomatis C., Ponti A., Cherqui D. (2009). The Learning curve in laparoscopic liver resection improved feasibility and reproducibility. Ann. Surg..

[15] Nomi T., Fuks D., Kawaguchi Y., Mal F., Nakajima Y., Gayet B. (2015). Learning curve for laparoscopic major hepatectomy. BJS.

[16] Wakabayashi G., Cherqui D., Geller D.A., Buell J.F., Kaneko H., Han H.S. (2015). Recommendations for laparoscopic liver resection a report from the second international consensus conference held in Morioka. Ann. Surg..

[17] Chen Y.W., Huang M.T., Chang T.C. (2019). Long term outcomes of simultaneou s laparoscopic versus open resection for colorectal cancer with synchronous liver metastases. Asian J. Surg..

[18] Wakabayashi G., Cherqui D., Geller D.A., Han H.S., Kaneko, Buell J.F. (2014). Laparoscopic hepatectomy is theoretically better than openhepatectomy: preparing for the 2nd international consensus conference on laparoscopic liver resection. J Hepatobiliary Pancreat Sci.

[19] Beppu T., Go Wakabayashi, Hasegawa K., Gotohda N., Mizuguchi T., Takahashi Y. (2015). Long-term and perioperative outcomes of laparoscopic versus open liver resection for colorectal liver metastases with propensity score matching: a multi- institutional Japanese study. J Hepatobiliary Pancreat Sci.

[20] Hasegawa Y., Nitta H., Sasaki A., Takahara T., Itabashi H., Katagiri H. (2015). Long- term outcomes of laparoscopic versus open liver resection for livermetastases from colorectal cancer: a comparative analysis of 168 consecutive cases at a single center. Surgery.

[21] Guo Y., Gao Y., Chen G., Li C., Dong G. (2018). Minimally Invasive versus open simultaneous resections of colorectal cancer and synchronous liver metastases: a Meta-Analysis. Am. Surg..

[22] Takasu C., Shimada M., Sato H., Miyatani T., Imura S., Morine Y. (2014). Benefits of simultaneous laparoscopic resection of primary colorectal cancer and liver metastases. Asian J. Endosc. Surg..

[23] Spampinato M.G., Mandala L., Quarta G., Del Medico P., Baldazzi G. (2012). One-stage, totally laparoscopic major hepatectomy and colectomy for colorectal neoplasm with synchronous liver metastasis: safety, feasibility and short-term outcome. Surgery.

[24] Tranchart H., Diop P.S., Lainas P., Pourcher G., Catherine L., Franco D., Dagher I. (2011). Laparoscopic major hepatectomy can be safely performed with colorectal surgery for synchronous colorectal liver metastasis. HPB.

